# Salvianolic Acid B Alleviates High Glucose-Induced Vascular Smooth Muscle Cell Inflammation by Upregulating the miR-486a-5p Expression

**DOI:** 10.1155/2024/4121166

**Published:** 2024-02-16

**Authors:** Man-Li Zhang, Man-Na Zhang, Hui Chen, Xia Wang, Kun Zhao, Xuan Li, Xuan Song, Fei Tong

**Affiliations:** ^1^Department of Critical Care Medicine, The Second Hospital of Hebei Medical University, 215 Heping West Road, Shijiazhuang, Hebei 050000, China; ^2^Department of Clinical Laboratory, The Second Hospital of Hebei Medical University, 215 Heping West Road, Shijiazhuang, Hebei 050000, China

## Abstract

The macrovascular complications of diabetes cause high mortality and disability in patients with type 2 diabetes mellitus (T2DM). The inflammatory response of vascular smooth muscle cell (VSMC) runs through its pathophysiological process. Salvianolic acid B (Sal B) exhibits beneficial effects on the cardiovascular system. However, its role and mechanism in diabetic vascular inflammatory response remain unclear. In this study, we found that Sal B reduced vascular inflammation in diabetic mice and high glucose- (HG-) induced VSMC inflammation. Subsequently, we found that Sal B reduced HG-induced VSMC inflammation by downregulating FOXO1. Furthermore, miR-486a-5p expression was obviously reduced in HG-treated VSMC. Sal B attenuated HG-induced VSMC inflammation by upregulating miR-486a-5p. Loss- and gain-of-function experiments had proven that the transfection of the miR-486a-5p mimic inhibited HG-induced VSMC inflammation whereas that of the miR-486a-5p inhibitor promoted HG-induced VSMC inflammation, thereby leading to the amelioration of vascular inflammation in the diabetic mice. Furthermore, studies had shown that miR-486a-5p inhibited FOXO1 expression by directly targeting its 3′-UTR. In conclusion, Sal B alleviates the inflammatory response of VSMC by upregulating miR-486a-5p and aggravating its inhibition of FOXO1 expression. Sal B exerts a significant anti-inflammatory effect in HG-induced VSMC inflammation by modulating the miR-486a-5p/FOXO1 axis.

## 1. Introduction

Diabetic macrovascular complications, including coronary artery disease, cerebrovascular disease, and peripheral vascular disease, lead to high mortality and morbidity in patients with type 2 diabetes mellitus (T2DM) [1]. The pathological basis of diabetic macrovascular complications is atherosclerosis, and the common basis of T2DM and atherosclerosis is low-grade, chronic, and subclinical inflammation. Inflammation is involved in the formation, development, rupture, and subsequent clinical acute events of diabetic atherosclerotic plaques [2]. Therefore, early anti-inflammatory therapy is an important intervention in the progression of macrovascular disease in T2DM. Vascular smooth muscle cell (VSMC) is the target cell for various cytokines and vasoactive substances. Experimental studies have shown that hyperglycemia exacerbates the inflammation of VSMC, which are the central link in the study of diabetic macrovascular disease [3]. Therefore, the effective control of VSMC inflammation in diabetic patients is crucial for the treatment of diabetic macrovascular disease.

Forkhead box transcription factor O1 (FOXO1) is associated with stress, apoptosis, glucose metabolism, angiogenesis, tumorigenesis, diabetes, and immune system diseases. Studies have confirmed that when FOXO1 in macrophages is stimulated by the inflammatory factors, the extracellular metastasis and phosphorylation state of the protein are disturbed, resulting in binding to the inflammatory factor IL-1*β* promoter, enhancing the expression of IL-1*β*, and promoting the occurrence of inflammatory responses [4]. However, the involvement of FOXO1 in high glucose- (HG-) induced VSMC inflammation needs to be investigated further.

Multiple miRNAs are involved in regulating gene expressions related to VSMC migration, inflammation, and proliferation in diabetic vascular remodeling, including miR-200b, miR-146a, miR-126, and miR-24 [5, 6]. Zhang et al. [7] demonstrated that in mouse podocytes, SC5B-9 regulates apoptosis by regulating the Kcnq1ot1/miR-486a-3p/NLRP3 axis. Sun et al. [8] showed that exercise could regulate the miR-486a-5p/Mst1 pathway to lower collagen deposition, inhibit myocardial cell apoptosis, and reduce cardiac remodeling in diabetic cardiomyopathy mice. Furthermore, the influence of miR-486a-5p on HG-induced VSMC inflammation needs to be investigated further.

*Salvia miltiorrhiza* is one of the most widely used traditional Chinese medicines, and its preparations are used to treat various cardiovascular diseases, such as coronary heart disease, ischemia–reperfusion, and hypertension. Among the active ingredients of *S. miltiorrhiza*, Salvianolic acid B (Sal B) has the highest content of water-soluble active ingredients. Sal B has anti-liver fibrosis effects, alleviates kidney injury, prevents cardiovascular diseases, and protects the nervous system, with cardiovascular disease prevention being the most prominent [9, 10]. Zhao et al. [11] showed that Sal B suppressed intimal hyperplasia and VSMC proliferation by downregulating the expression of miR-146a. Despite our progress in the mechanisms by which Sal B protects the cardiovascular system, it is still not determined whether miRNAs mediate the effects of Sal B on VSMC inflammation in T2DM.

This study focuses on diabetic mice and VSMC to investigate how Sal B inhibits the inflammatory response of VSMC in diabetic mice by adjusting miR-486a-5p expression.

## 2. Materials and Methods

### 2.1. Animal Models

All animals were raised and cared for in accordance with the regulations of the Institutional Animal Care and Use Committee of Hebei Medical University. In this study, 6-to 8-week-old male C57BL/6 mice were randomly classified as the control group (NC), diabetes group (DM), and diabetes + Sal B group (DM + Sal B*; n* = 7 for each group). The mice model of T2DM was established by a high-calorie diet and multiple intraperitoneal injections of streptozotocin (STZ, Enzo). The mice were fed with a high-calorie diet (≥15% crude protein content, ≥12% ether extract, and 4,000 kcal/kg of total calories) for 4 weeks and then intraperitoneally injected with STZ (50 mg/kg) for 2–4 consecutive days (if the fasting blood glucose of mice did not exceed 7.0 mM, it was recommended to supplement STZ injection in time, up to 4 times). The control mice were fed with standard caloric mouse feed and injected with citrate buffer. General conditions, such as the water intake and body weight of the mice, were observed. The blood glucose level of the mice increased 5–7 days after modeling, and the mice with a blood glucose level of >16.7 mM were diagnosed with diabetes. The mice in the Sal B treatment group were intraperitoneally injected with Sal B (7 mg/kg/day; MCE, purity 99.88%) when they were given a high-calorie diet, which lasted for 16 weeks. Animals that did not receive Sal B were injected intraperitoneally with normal saline. After STZ injection for 12 weeks, all mice were euthanized, and the thoracic and abdominal aortas were harvested to detect miRNA, mRNA, and protein expressions.

### 2.2. Cell Culture and Treatment

Mouse aortic VSMCs were purchased from ATCC (No. CRL2797™) and cultured in low-glucose (LG) Dulbecco's modified Eagle's medium (DMEM, Gibco Life Technologies) containing 100 *μ*g/ml of streptomycin and 100 units/ml of penicillin. After 80% of the cells were fuzed, the subculture was passaged at a ratio of 1 : 4 every 4 days. The VSMCs were stimulated with LG (5.5 mM), HG (25 mM), or HG (25 mM) plus different concentrations of Sal B (2.5, 5, and 10 *μ*M) for 24 hr. Moreover, the VSMCs were treated with HG (25 mM) plus 10 *μ*M Sal B for 6, 12, or 24 hr. Then, 293A cells (ATCC, No. CTL-1573) were cultured in DMEM containing 10% FBS.

### 2.3. ELISA

The plasma concentration of IL-1*β* (KE10003, Proteintech) or TNF-*α* (KE10002, Proteintech) was detected in accordance with the instructions for commercial ELISA kits. The absorbance at 450 nm was read using a microtiter plate reader (SPECTRAFluor Plus, Tecan).

### 2.4. Isolation of RNA and Real-Time PCR

According to the manufacturer's protocol, total RNA was extracted using the QIAzol cleavage reagent and the quality of RNA should be determined using the BioSpectrometer (Thermo NanoDrop 2000). When analyzing the microRNA, reverse transcription and qRT-PCR were performed using the miScript II RT kit (QIAGEN GmbH) and the miScript SYBR Green PCR kit, respectively. The internal control was RNU6b (U6). When analyzing the mRNA, the total RNA was reverse-transcribed to first-strand cDNA using the M-MLV First-Strand Kit (Life Technologies) following the manufacturer's protocol. Real-time PCR analysis was conducted using the Platinum SYBR Green qPCR SuperMix UDG Kit (Invitrogen) and the ABI 7500 FAST System. The internal control was GAPDH. The relative expression of transcripts was calculated using 2^−*ΔΔ*Ct^. All PCRs were repeated three times. Table S1 shows the primer sequences.

### 2.5. Western Blot Analysis

Proteins from blood vessels or cells were prepared with lysis buffer (1% Triton X-100, 150 mM NaCl, 10 mM Tris-HCl, pH 7.4, 1 mM EDTA, 1 mM EGTA, pH 8.0, 0.2 mM Na_3_VO_4_, 0.2 mM phenylmethylsulfonyl fluoride, and 0.5% NP-40). Equal amounts of protein were separated by SDS-PAGE and transferred to the PVDF membrane (Millipore). After blocking the membrane with 5% milk in TTBS for 2 hr at 37°C, incubated with the primary antibody overnight at 4°C: anti-IL-1*β* (1 : 1,000, ab9722, Abcam), anti-TNF-*α* (1 : 1,000, ab183218, Abcam), anti-FOXO1 (1 : 1,000, ab52857, Abcam), and anti-*β*-actin (1 : 1,000, ab8227, Abcam). On the second day, washed the membrane and incubated it with the secondary antibody for 1 hr at room temperature, and then evaluated with the ECL detection system.

### 2.6. Transfection and Plasmid Constructs

According to the manufacturer's protocol, use Lipofectamine 2000 (Invitrogen) transfected cells. Order miR-486a-5p mimics/inhibitors and their respective controls from GenePharma Co. Ltd. After transfection for 24 hr, harvest the cells and lyse them for the subsequent experiments. The luciferase reporter plasmid was constructed by the restriction-enzyme digestion and one-step cloning (ClonExpress II One-Step Cloning Kit, C112-02; Vazyme Biotech Co., Ltd.). The 3′-UTR sequences of FOXO1 containing the miR-486a-5p target site (wild type or mutant) were inserted into the pmir-GLO Dual-Luciferase miRNA Target Expression Vector (Promega Corp.) digested by Xho1 and Sal1. Table S2 shows the 3′-UTR of FOXO1 contain miR-486a-5p target site or its mutated sequences.

### 2.7. Target Prediction

The potential target microRNAs of FOXO1 were predicted using the following algorithms: RNAhybrid (http://bibiserv.techfak.uni-bielefeld.de/rnahybrid/submission.html) and miRanda (https://www.microrna.org) [12, 13].

### 2.8. Luciferase Assays

Here, 293A cells were seeded into 24-well plates. miR-486a-5p mimic (or mimic-control) or miR-486a-5p inhibitor (or inhibitor-control) was cotransfected with FOXO1 reporter constructs (wild type or mutant) or empty vectors. After transfection for 24 hr, the cells were then harvested for the subsequent experiments. According to the manufacturer's protocol, the luciferase activity was measured using the Dual-Glo Luciferase Assay System (Promega). Firefly luciferase (FLuc) activity was measured and normalized with renal luciferase (RLuc) activity.

### 2.9. Statistical Analysis

All values were expressed as mean ± SEM. The differences between the two groups were analyzed using the Student's *t*-test or analysis of variance. In all analyses, *P*  < 0.05 was considered significant. Each group of the experiments was repeated at least three times independently.

## 3. Results

### 3.1. Sal B Attenuates Vascular Inflammation in Diabetic Mice

**Figure 1:**
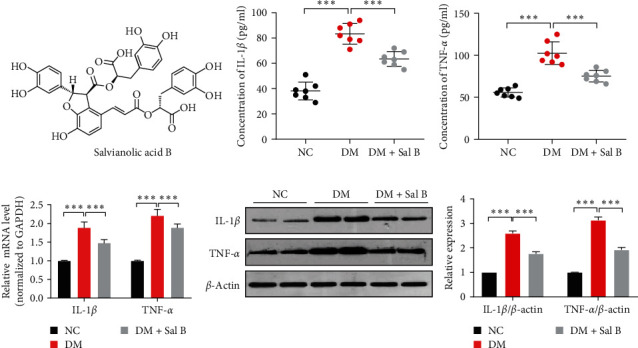
Sal B attenuates vascular inflammation in diabetic mice. (a) The chemical structure of Sal B. (b, c) The concentrations of IL-1*β* and TNF-*α* in mice serum were detected by ELISA.  ^*∗∗∗*^*P* < 0.001 vs. DM group (*n* = 7). (d) qRT-PCR measured the IL-1*β* and TNF-*α* expression in mice aortic vessels. Relative mRNA expression was presented after normalizing to GAPDH (means ± SEM, *n* = 7).  ^*∗∗∗*^*P* < 0.001 vs. DM group. (e) The IL-1*β* and TNF-*α* expression in mice aortic vessels was measured by western blot analysis (left panel). Bar graphs show the relative level of these proteins for three independent experiments.  ^*∗∗∗*^*P* < 0.001 vs. DM group (right panel, *n* = 7).

Sal B, a condensate of one molecule of caffeic acid and three molecules of Danshensu (Figure 1(a)), is one of the most studied salvianolic acids. We injected Sal B intraperitoneally into T2DM mice to observe its effect on vascular inflammation. The ELISA results indicated that the concentration of the proinflammatory cytokine IL-1*β* and TNF-*α* in diabetic mice serum was significantly onefold higher than that of nondiabetic mice and Sal B abated the levels of the inflammatory factors in the serum of diabetic mice (Figures 1(b) and 1(c)). Subsequently, qRT-PCR (Figure 1(d)) and western blot analysis (Figure 1(e)) exhibited that, compared with the nondiabetic mice, the inflammatory factor expression in the thoracic and abdominal aorta of diabetic mice was obviously higher and Sal B could decrease the inflammation factor expression. Overall, Sal B inhibits vascular inflammation in the diabetic mice.

### 3.2. Sal B Reduces HG-Induced VSMC Inflammation

**Figure 2:**
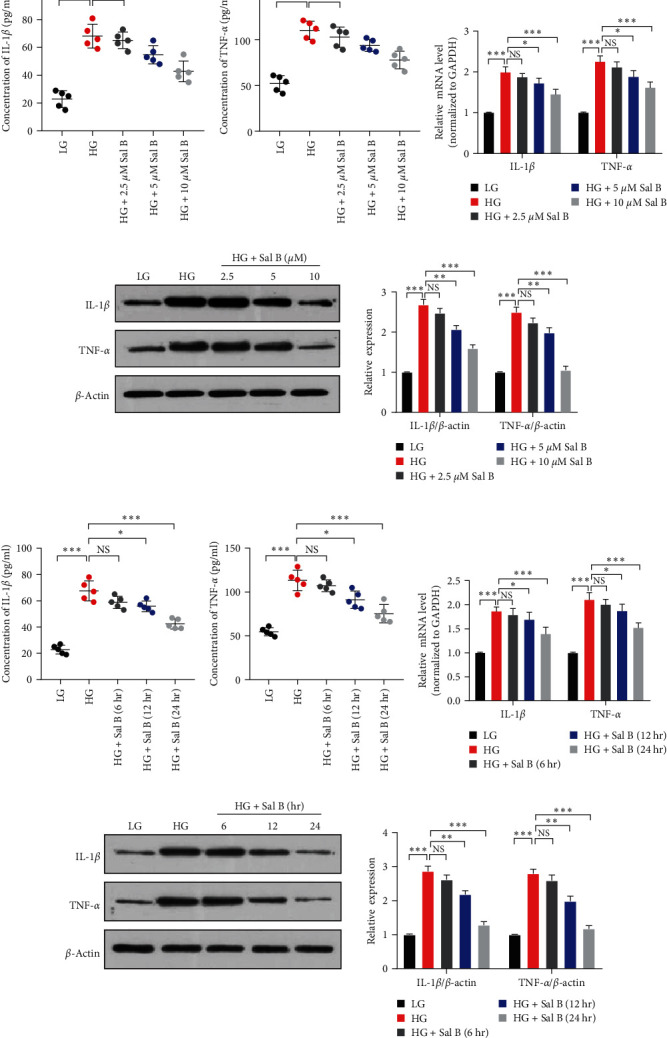
Sal B reduces HG-induced VSMC inflammation. (a, b) VSMCs were incubated with LG (5.5 mM), HG (25 mM), and HG (25 mM) plus different concentrations of Sal B (2.5, 5, and 10 *μ*M) for 24 hr. ELISA detected the IL-1*β* and TNF-*α* concentrations of the culture medium.  ^*∗*^*P* < 0.05 and  ^*∗∗∗*^*P* < 0.001 vs. HG group (*n* = 5). (c) VSMCs were stimulated as in 2(a). Relative expression of IL-1*β* and TNF-*α* mRNA was examined by qRT-PCR and presented after normalizing to GAPDH (means ± SEM, *n* = 3).  ^*∗*^*P* < 0.05 and  ^*∗∗∗*^*P* < 0.001 vs. HG group. (d) VSMCs were incubated as in 2(a). The protein expression of IL-1*β* and TNF-*α* was detected by western blotting (left panel). Bar graphs show the relative level of these proteins, which were repeated three times.  ^*∗∗*^*P* < 0.01 and  ^*∗∗∗*^*P* < 0.001 vs. HG group (right panel, *n* = 3). (e, f) The VSMCs were cultured with LG (5.5 mM), HG (25 mM), and HG (25 mM) plus Sal B (10 *μ*M) for 6, 12, and 24 hr. ELISA measured the IL-1*β* and TNF-*α* concentrations of the culture medium.  ^*∗*^*P* < 0.05 and  ^*∗∗∗*^*P* < 0.001 vs. HG group (*n* = 5). (g, h) The VSMCs were stimulated as in 2(e). Relative expression of IL-1*β* and TNF-*α* mRNA was examined by qRT-PCR and presented after normalizing to GAPDH (means ± SEM, *n* = 3). The protein expression of IL-1*β* and TNF-*α* was detected by western blotting (left panel).  ^*∗*^*P* < 0.05,  ^*∗∗*^*P* < 0.01, and  ^*∗∗∗*^*P* < 0.001 vs. HG group *n* = 3.

Because diabetic macrovascular disease is associated with abnormal VSMC proliferation and inflammation, we examined whether the effect of Sal B in inhibiting vascular inflammation in diabetic mice is related to HG-induced VSMC inflammation. As shown in Figures 2(a) and 2(b), after incubating VSMC with HG, the levels of IL-1*β* and TNF-*α* in the culture medium increased. However, Sal B reduced the levels of proinflammatory factors in a dose-dependent manner induced by HG. Furthermore, at the mRNA and protein levels, HG increased the proinflammatory factor expression and Sal B ameliorated the proinflammatory effects of HG (Figures 2(c) and 2(d)). The optimal concentration of Sal B was 10 *μ*M. The following results showed that the levels of HG-induced proinflammatory factors were downregulated by Sal B in a time-dependent manner, and the optimal time was 24 hr (Figure 2(e)–2(h)). These results clearly indicated that Sal B reduces HG-induced VSMC inflammation.

### 3.3. Sal B Inhibits HG-Induced VSMC Inflammation by Downregulating FOXO1

**Figure 3:**
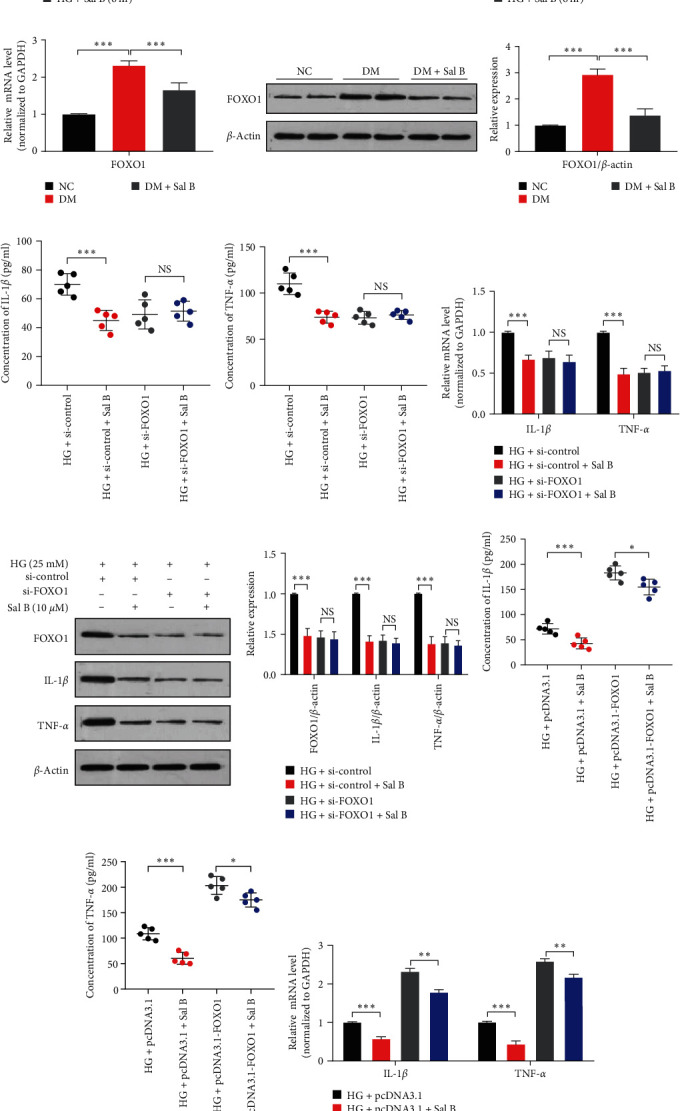
Sal B inhibits HG-induced VSMC inflammation by downregulating FOXO1. (a–d) The VSMCs were treated as in 2(a) or 2(e). qRT-PCR and western blot analysis measured the expression of FOXO1.  ^*∗*^*P* < 0.05,  ^*∗∗*^*P* < 0.01, and  ^*∗∗∗*^*P* < 0.001 vs. HG group (*n* = 3). (e, f) The FOXO1 expression in the mouse aortic vessels was measured by qRT-PCR and western blot analysis.  ^*∗∗∗*^*P* < 0.001 vs. DM group (*n* = 7). (g, h) The VSMCs were transfected with si-FOXO1 or si-control for 24 hr and then incubated with HG (25 mM) plus or without Sal B (10 *μ*M). ELISA measured the IL-1*β* and TNF-*α* concentrations in the culture medium.  ^*∗∗∗*^*P* < 0.001 vs. each control group (*n* = 5). (i, j) The VSMCs were treated as in 3(g), and qRT-PCR and western blot analysis measured the expression of IL-1*β* and TNF-*α* in VSMC.  ^*∗∗∗*^*P* < 0.001 vs. each control group (*n* = 3). (k, l) VSMCs were transfected with pcDNA3.1 or pcDNA3.1-FOXO1 for 24 hr and then treated with HG (25 mM) plus or without Sal B (10 *μ*M). The expression levels of IL-1*β* and TNF-*α* were detected by ELISA.  ^*∗*^*P* < 0.05 and  ^*∗∗∗*^*P* < 0.001 vs. each control group (*n* = 5). (m, n) The VSMCs were treated as in 3(k). The expression levels of IL-1*β*, TNF-*α*, and FOXO1 were measured by qRT-PCR and western blot analysis.  ^*∗∗*^*P* < 0.01 and  ^*∗∗∗*^*P* < 0.001 vs. each control group (*n* = 3).

FOXO1 is involved in cell proliferation, apoptosis, inflammation, migration, metabolism, and other functions. Therefore, we aimed to examine whether FOXO1 plays a key role in the anti-inflammatory effect of Sal B. HG upregulated the FOXO1 expression, and the addition of Sal B to HG-treated VSMC led to a dose-dependent decrease of FOXO1, reducing the mRNA level of FOXO1 to ∼60% (Figures 3(a) and 3(b)). Subsequent results showed that Sal B reduced the FOXO1 expression in a time-dependent manner, with this inhibitory effect being most pronounced after exposure to Sal B 24 hr (Figures 3(c) and 3(d)). Next, we examined the FOXO1 expression in the blood vessels of diabetic mice treated with Sal B. Figures 3(e) and 3(f) showed that Sal B reduced the FOXO1 expression in the blood vessels of diabetic mice. To determine whether Sal B reduces inflammation in VSMC through FOXO1, we applied small interfering RNAs to knock down FOXO1. As shown in Figures 3(g) and 3(h), after knocking down FOXO1, the level of inflammatory factors in the medium was significantly reduced. At this time, the VSMCs were stimulated with Sal B, and the expression level of inflammatory factors did not change significantly. Consistent with these findings, at the mRNA and protein levels, no significant change was observed in the inflammatory factors after knocking down FOXO1, regardless of whether Sal B was administered or not (Figures 3(i) and 3(j)). To further investigate whether Sal B inhibits HG-induced VSMC inflammation by FOXO1, we overexpressed FOXO1 by transfecting VSMC with pcDNA 3.1-FOXO1 and then treated VSMC with HG or HG plus Sal B. In the medium of VSMC, Sal B significantly reduced HG-induced IL-1*β* and TNF-*α* expression in pcDNA 3.1 transfected cells, while inhibition of Sal B was weakened in pcDNA 3.1-FOXO1 transfected cells (Figures 3(k) and 3(l)). The same results were obtained by qRT-PCR and western blot analysis (Figures 3(m) and 3(n)), suggesting that Sal B inhibits HG-induced VSMC inflammation by downregulating FOXO1. So to sum up, Sal B inhibits HG-induced VSMC inflammation through FOXO1.

### 3.4. Sal B Downregulates HG-Induced VSMC Inflammation by Upregulating miR-486 a-5p

**Figure 4:**
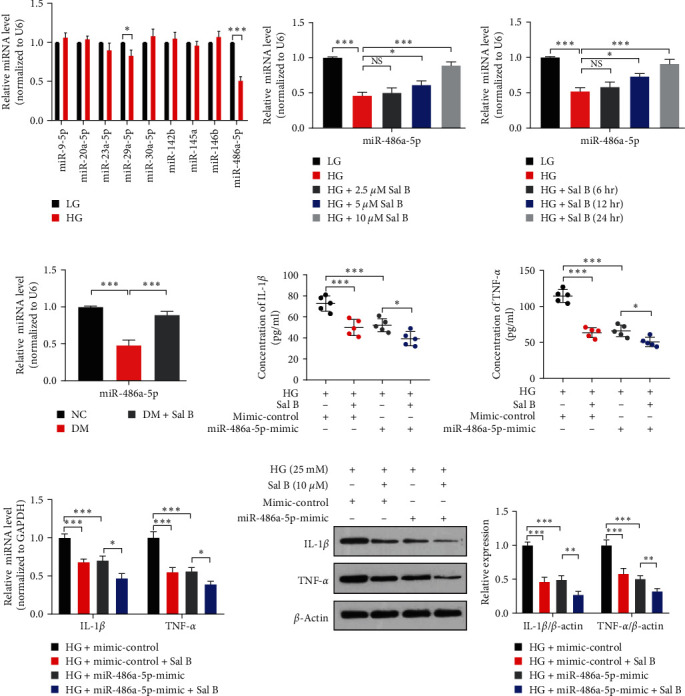
Sal B downregulates HG-induced VSMC inflammation by upregulating miR-486a-5p. (a) The VSMCs were treated with LG (5.5 mM) or HG (25 mM) for 24 hr, and qRT-PCR detected the miRNA expression.  ^*∗*^*P* < 0.05 and  ^*∗∗∗*^*P* < 0.001 vs. LG group (*n* = 3). (b, c) The VSMCs were treated as in 2(a) or 2(e). qRT-PCR measured the miR-486a-5p expression.  ^*∗*^*P* < 0.05 and  ^*∗∗∗*^*P* < 0.001 vs. HG group (*n* = 3). (d) qRT-PCR measured the miR-486a-5p expression in the mouse aortic vessels.  ^*∗∗∗*^*P* < 0.001 vs. DM group (*n* = 7). (e, f) The VSMCs were transfected with mimic-control or miR-486a-5p mimic for 24 hr and then treated with HG (25 mM) plus or without Sal B (10 *μ*M). ELISA measured the IL-1*β* and TNF-*α* concentrations of the culture medium.  ^*∗*^*P* < 0.05 and  ^*∗∗∗*^*P* < 0.001 vs. each control group (*n* = 5). (g, h) The VSMCs were cultured as in 4(e). qRT-PCR and western blot analysis measured the IL-1*β* and TNF-*α* expression.  ^*∗*^*P* < 0.05,  ^*∗∗*^*P* < 0.01, and  ^*∗∗∗*^*P* < 0.001 vs. each control group (*n* = 3).

The findings of this study also raise a question as to how FOXO1 expression is upregulated in HG-treated VSMC. Because miRNAs can regulate many gene expressions at the posttranscriptional level, we used the miRanda and TargetScan target prediction programs to find miRNAs that might target FOXO1 3′-UTR and detected nine miRNAs. In HG-treated VSMC, only miR-29a-5p and miR-486a-5p were reduced, with miR-486a-5p being the most significantly downregulated (Figure 4(a)). Next, we investigated whether Sal B had an effect on miR-486a-5p expression in HG-stimulated VSMC. The qRT-PCR revealed that Sal B upregulated the miR-486a-5p expression in a dose- and time-dependent manner. The most significant effect was 10 *µ*M Sal B treatment for 24 hr (Figures 4(b) and 4(c)). In the abdominal aorta tissue of mice, the miR-486a-5p expression in the blood vessels of diabetic mice was downregulated compared with that of nondiabetic mice, and Sal B could upregulate the miR-486a-5p expression in the blood vessels of diabetic mice (Figure 4(d)).

To further confirm whether miR-486a-5p has an effect on HG-induced VSMC inflammation, we transfected the VSMC with miR-486a-5p mimic to observe the inflammatory gene expression. As shown in Figures 4(e) and 4(f), after the VSMC were transfected with miR-486a-5p mimic, the concentration of the inflammatory factors in the medium was significantly reduced, and after stimulating the cells with Sal B, the concentration of inflammatory factors was slightly reduced. Furthermore, the miR-486a-5p mimic reduced the mRNA and protein expression levels of IL-1*β* and TNF-*α*. Sal B only slightly reduced the inflammatory factors after the transfection of the miR-486a-5p mimic (Figures 4(g) and 4(h)). Overall, Sal B reduces HG-induced VSMC inflammation via miR-486a-5p.

### 3.5. miR-486a-5p Targets FOXO1

**Figure 5:**
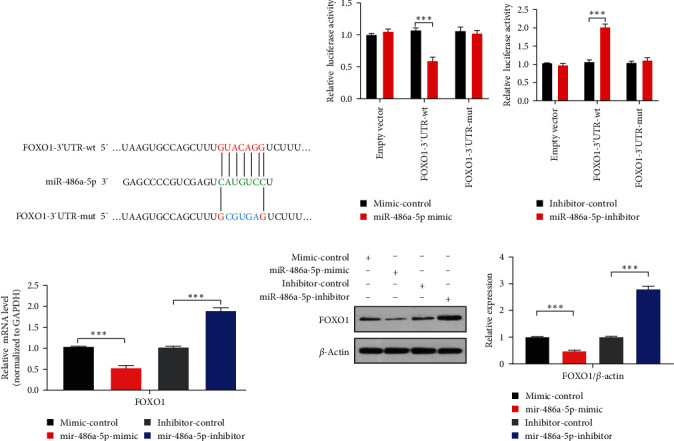
miR-486a-5p targets FOXO1. (a) miR-486a-5p binding site in the 3′-UTR of FOXO1 mRNA (green). Blue is the mutation position. (b, c) Luciferase reporter assays in 293A cells were cotransfected with the constructs containing wt or mutant (mut) FOXO1 3′-UTR and miR-486a-5p mimic/inhibitor or mimic-control/inhibitor-control. Experiments were performed in triplicate and each value is the mean ± SEM of three independent experiments.  ^*∗∗∗*^*P* < 0.001 vs. mimic-control or inhibitor-control group (*n* = 3). (d, e) VSMCs were transfected with indicated constructs for 24 hr, and qRT-PCR or western blot detected FOXO1 expression (left panel).  ^*∗∗∗*^*P* < 0.001 vs. mimic-control or inhibitor-control group (*n* = 3).

Next, we searched for potential matching check points for miR-486a-5p in FOXO1 3′-UTR with computer-based sequence analysis (TargetScan and miRanda). A putative miR-486a-5p binding check point in FOXO1 3′-UTR was found (Figure 5(a)). Considering that FOXO1 is the predicted target of miR-486a-5p, we detected whether miR-486a-5p affects the expression of FOXO1. To this end, we applied miR-486a-5p mimics or inhibitors to transfect 293A cells. 293A cells were cotransfected with miR-486a-5p mimics and FOXO1 3′-UTR-luciferase reporter gene containing miR-486a-5p binding checkpoint, type (wt) or mutation (mut). The dual luciferase reporter assay revealed that the luciferase activity driven by wt-FOXO1 3′-UTR was significantly reduced when the cells were transfected with miR-486a-5p mimic. The difference is that the mutations at the miR-486a-5p binding site in FOXO1 3′-UTR counteracted this inhibition (Figure 5(b)). By contrast, cells cotransfected with miR-486a-5p inhibitors obviously enhanced the luciferase activity driven by wt-FOXO1 3′-UTR, but miR-486a-5p was binding to the check point in FOXO1 3′-UTR. The mutation in the FOXO1 3′-UTR eliminated the enhanced activity of luciferase (Figure 5(c)). We further transfected the VSMC with miR-486a-5p mimics or inhibitors and measured the FOXO1 expression. As shown in Figures 5(d) and 5(e), the transfection of miR-486a-5p mimic reduced the FOXO1 expression, and the inhibitors of miR-486a-5p increased the FOXO1 expression. Overall, miR-486a-5p targets FOXO1.

## 4. Discussion

T2DM is a natural immune and chronic inflammatory disease. Diabetic macrovascular disease seriously affects the quality of life of patients with diabetes. The control of blood glucose alone can significantly improve the microvascular complications of diabetes but not the macrovascular complications caused by atherosclerosis. Inflammation runs through the whole process of the formation and development of atherosclerotic plaques in the large blood vessels of patients with T2DM [14]. Several studies have shown that hyperglycemia can promote the inflammation in VSMC [15]. Therefore, the inhibition of the inflammatory response of VSMC has become an effective treatment strategy for diabetic macroangiopathy. The main findings of this study are as follows: (1) Sal B alleviates the vascular inflammation in diabetic mice; (2) Sal B reduces the HG-induced proinflammatory gene expression in VSMC; (3) Sal B inhibits HG-induced the VSMC inflammation by downregulating FOXO1; (4) Sal B downregulates the VSMC inflammation induced by HG through upregulating miR-486a-5p; and (5) Sal B attenuates the HG-induced VSMC inflammation by regulating the miR-486a-5p/FOXO1 axis.

Sal B is a water-soluble active ingredient extracted from the roots and rhizomes of *S. miltiorrhiza*. Sal B can alleviate myocardial ischemia–reperfusion injury, resist myocardial cell injury, protect vascular endothelial cells, inhibit VSMC proliferation, prevent atherosclerosis, exhibit antitumor effects, and so on. In the rat intestinal epithelial cells, Sal B ameliorated the oxidative stress-induced mitochondrial dysfunction and intestinal epithelial barrier dysfunction by activating the Akt/GSK3*β* signaling pathway [16]. Li et al. [17] reported that in the cardiovascular system, Sal B reduced IGFBP3 induced by HG, enhanced cell proliferation, and promoted angiogenesis. Moreover, Sal B inhibits balloon angioplasty-induced neointimal hyperplasia and VSMC migration and proliferation by restraining the CXCR4 receptor expression [18]. In spite of this, it is not yet determined whether Sal B can play a protective role in the blood vessels by inhibiting VSMC inflammation. In this study, Sal B decreased the concentration of proinflammatory cytokines in diabetic mice plasma and restrained inflammatory cytokine expressions in the blood vessels of diabetic mice.

miRNAs are small endogenous RNAs that regulate gene expressions at the posttranscriptional level. Its abnormal expression is closely linked to a variety of diseases, including cardiovascular diseases, diabetes, rheumatoid arthritis, and cancer. In diabetic nephropathy, CASC2 reduces HG-induced podocyte injury by mediating the miR-9-5p/PPAR*γ* axis [19]. A study has shown that Sal B ameliorates the blood–brain barrier dysfunction induced by HG through regulating the miR-200b and VEGF signaling pathways [20]. Sal B inhibits the epithelial–mesenchymal transformation of the hepatic stellate cells by regulating the miR-152/DNMT1 axis; thus, inhibiting liver fibrosis [21]. In human rheumatoid fibroblast-like synovial cells (MH7A), Sal B exerts antiapoptotic and anti-inflammatory effects through the upregulation of miR-142-3p [22]. Zhao et al. [11] reported that Sal B inhibited intimal hyperplasia and reduced VSMC proliferation induced by Ang II through downregulating the expression of miR-146a; thus, playing a protective role in the blood vessels. In this study, Sal B attenuated HG-induced VSMC inflammation by upregulating miR-486a-5p.

FOXO1 changes with the cell stimulation to maintain cellular homeostasis during pathophysiological processes, such as oxidative stress, cell proliferation, autophagy, and apoptosis. FOXO1 can regulate the expression of cell cycle arrest genes, anti-oxidant enzymes, apoptosis- and autophagy-related genes, and metabolism and immune regulatory factors [23]. Studies have shown that FOXO1 is strongly associated with diabetes. In cardiac fibroblasts (CFs), HG induced the phenotypic transformation of CFs via FOXO1, thereby disrupting extracellular matrix (ECM) homeostasis [24]. In the HG environment, Ski promoted fibroblast proliferation and inhibited apoptosis through the FOXO1 pathway [25]. A study has shown that metformin inhibited the HG-induced mesangial cell ECM, inflammation, and proliferation by regulating the SIRT1/FOXO1 axis [26]. In this study, we had proven that HG upregulated the FOXO1 expression and Sal B counteracted this effect. Further mechanistic studies had shown that FOXO1 was the target gene of miR-486a-5p. Moreover, Sal B inhibited VSMC inflammation by regulating the miR-486a-5p/FOXO1 axis.

Sal B can play a protective role in blood vessels through multiple pathways and multiple targets. Lin et al. [27] have shown that Sal B significantly reverses Ang II-induced arterial blood pressure by decreasing the expression of AT1 receptor and NADPH oxidase in human umbilical vein endothelial cells (HUVEC). Sal B alleviates hydrogen peroxide (H_2_O_2_)-induced oxidative stress in HUVEC by activating AMPK pathway and downregulating mTOR pathway to promote autophagy [28]. Tang et al. [29] have demonstrated that Sal B inhibits endoplasmic reticulum (ER) stress and downregulated TXNIP by regulating AMPK/FoxO4/KLF2 and Syndecan-4/Rac1/ATF2 signaling pathways, and inhibits NLRP3 inflammasome activation by downregulating NLRP3 and cleaved caspase-1. This results in decreased secretion of IL-18 and IL-1*β*, which ultimately alleviating endothelial damage caused by ER stress [29]. Studies have shown that Sal B improves autophagy dysfunction by inhibiting the Akt/mTOR signaling pathway and reduces cholesterol crystal- (CHCs-) induced macrophage apoptosis [30]. This study suggests that Sal B inhibits HG-induced vascular inflammation through the miR-486a-5p/FOXO1 pathway. However, there are some limitations to this study. First, we only investigated the effect of Sal B on VSMC inflammation, however, whether and how Sal B regulates the VSMC proliferation, oxidative stress, or autophagy to protect blood vessels in an HG environment or diabetes remains to be further investigated. Second, we evaluated only one potential downstream target of miR-486a-5p, and further studies are needed to find other targets that may also contribute to the vascular protective effect of miR-486a-5p. Third, this study lacks further confirmation from the experiments in diabetic mice. Therefore, further clinical studies are needed to confirm the findings of this study.

## 5. Conclusions

In summary, our results indicated that Sal B can effectively ameliorate HG-induced VSMC inflammation and vascular inflammation in diabetic mice by upregulating the miR-486a-5p expression. Furthermore, the modulation of the miR-486a-5p/FOXO1 axis can alleviate the VSMC inflammation in T2DM.

## Data Availability

Data supporting the findings of this study are available from the corresponding author upon request.

## Supplementary Materials

Supplementary Materials Table S1: primers for real-time PCR. Table S2: 3′-UTR of FOXO1 contain miR-486a-5p target site or its mutated sequences.
