# Supplementary material for: Salvianolic Acid B Alleviates High Glucose-Induced Vascular Smooth Muscle Cell Inflammation by Upregulating the miR-486a-5p Expression
Source: Mediators Inflamm. 2024 Feb 16;2024:4121166. doi: 10.1155/2024/4121166 (PMC10890902; doi:10.1155/2024/4121166)
Supplement: Supplementary Materials — Table S1: primers for real-time PCR. Table S2: 3′-UTR of FOXO1 contain miR-486a-5p target site or its mutated sequences. [file 4121166.f1.docx]

**Salvianolic acid B alleviates high glucose-induced vascular smooth muscle cell inflammation by upregulating the miR-486a-5p expression**

Man-li Zhang^1^, Man-na Zhang^2^, Hui Chen^1^, Xia Wang^1^, Kun Zhao^1^, Xuan Li^1^, Xuan Song^1^, Fei Tong^1^*

1 Department of Critical Care Medicine, The Second Hospital of Hebei Medical University, 215 Heping West Road, Shijiazhuang, Hebei, 050000, China

2 Department of Clinical Laboratory, The Second Hospital of Hebei Medical University, 215 Heping West Road, Shijiazhuang, Hebei, 050000, China

*Correspondence should be addressed to Fei Tong; tongfei168@163.com

**Supplemental Table I**

Primers for real-time PCR

| **Primer** | **Forward** | **Reverse** |
| --- | --- | --- |
| GAPDH | AAGGTGAAGGTCGGAGTC | GATTTTGGAGGGATCTCG |
| IL-1β | CAACCAACAAGTGATATTCTCCATG | GATCCACACTCTCCAGCTGCA |
| TNF-α | CATCTTCTCAAAATTCGAGTGACAA | TGGGAGTAGACAAGGTACAACCC |
| FOXO1 | CTGGGTGTCAGGCTAAGAGT | GGGGTGAAGGGCATCTTT |

**Supplemental Table II**

3'UTR of FOXO1 contain miR-486a-5p target site or its mutated sequences

| **Gene** | **Sequences (5’-3’)** |
| --- | --- |
| FOXO1-wt-F: | GGAAAAAAAAAGATTAAATGCCAGCTTTGTACAGGTCTTTTCTATTTTTTTTTG |
| FOXO1-wt-R: | CAAAAAAAAATAGAAAAGACCTGTACAAAGCTGGCATTTAATCTTTTTTTTTCC |
| FOXO1-mut-F: | GGAAAAAAAAAGATTAAATGCCAGCTTTGCGTGAGTCTTTTCTATTTTTTTTTG |
| FOXO1-mut-R: | CAAAAAAAAATAGAAAAGACTCACGCAAAGCTGGCATTTAATCTTTTTTTTTCC |
